# The Link between Magnesium Supplements and Statin Medication in Dyslipidemic Patients

**DOI:** 10.3390/cimb45040205

**Published:** 2023-04-05

**Authors:** Roxana Nartea, Brindusa Ilinca Mitoiu, Ioana Ghiorghiu

**Affiliations:** 1 Clinical Department 9, Carol Davila University of Medicine and Pharmacy, 050474 Bucharest, Romania; 2 National Institute for Rehabilitation, Physical Medicine and Balneoclimatology, 030079 Bucharest, Romania; 3 Agrippa Ionescu Clinical Emergency Hospital, 077016 Bucharest, Romania

**Keywords:** magnesium supplements, metabolic syndrome, lipoproteins, statin medication, dyslipidemia

## Abstract

Many investigations have discovered a connection between statins and magnesium supplements. On one hand, increasing research suggests that chronic hypomagnesemia may be an important factor in the etiology of some metabolic illnesses, including obesity and overweight, insulin resistance and type 2 diabetes mellitus, hypertension, alterations in lipid metabolism, and low-grade inflammation. Chronic metabolic problems seem to be prevented by a high Mg intake combined with diet and/or supplements. On the other hand, it is known that statins lower the frequency of cardiac events, stroke, and mortality, not by lowering LDL-C, but by the capacity to reduce mevalonate formation. That will enhance endothelial function, inhibit vascular smooth muscle cell proliferation and migration and encourage macrophages to promote plaque stability and regression while reducing inflammation. Taking these factors into consideration, we did an extensive analysis of the relevant literature, comparing the effects of Mg^2^ and statin medications on lipoproteins and, implicitly, on the key enzymes involved in cholesterol metabolism.

## 1. Introduction

Dyslipidemia, an important factor in defining metabolic syndrome (MetS), represents a worldwide challenge, due to the increased risk of atherosclerosis and cardiovascular diseases (CVD) [1]. Dyslipidemia is characterized by elevated levels of total cholesterol and/or triglycerides, with decreased high-density lipoprotein (HDL) levels. Elevated levels of LDL (low-density lipoprotein) and VLDL (very low-density lipoprotein-density) can be also observed.

The progression to atherosclerosis depends on the vascular endothelial metabolism (Kruppel-like factor 2 expression) and LDL serum level (above 20–40 mg/dL) [2,3,4].

Statins are the main treatment for LDL cholesterol reduction because they demonstrably lower cardiovascular morbidity and mortality (according to International Guidelines for the Management of Dyslipidemia 2022), in cases of high-risk conditions, such as clinical atherosclerosis, abdominal aortic aneurysm, diabetes mellitus, chronic kidney disease (age ≥50 years), and patients with LDL-C ≥ 5.0 mmol/L. However, many studies highlight a series of adverse reactions and/or rather modest results in reducing the level of LDL cholesterol and indirectly reducing dyslipidemia, after statin administration [5,6].

There are several factors involved in abnormal lipid profiles such as genetic background, a Western-style diet, alcohol abuse, being overweight or obese, insulin resistance, or some chronic conditions, such as nephrotic syndrome [6,7,8,9,10]. Recent studies suggest that serum magnesium levels can be associated with lipid abnormalities (Table S1, see Supplementary Materials). As epidemiological research is refined by the description of the biological and pathophysiological mechanisms, the goal of this study is to summarize the current understanding of the pathological relationships between dyslipidemia, statin use, and serum magnesium levels.

It is not just theoretically interesting to learn about the connections between dyslipidemia and serum magnesium levels. Magnesium, through its action on lecithin–cholesterol acyl transferase (LCAT), can improve the metabolism of lipoproteins, and implicitly dyslipidemia [11,12,13,14]. If correctly controlled, interventions like diet, exercise, and magnesium supplementation could be very beneficial and lessen the effect of dyslipidemia on CVD. [14].

Although many studies in the scientific literature present the effects of statin therapy or magnesium supplementation in dyslipidemia, there are rather few articles to discuss the results of a combined treatment.

## 2. Literature Selection

We conducted a thorough review of the literature and included papers that discussed the role of dietary magnesium intake in the etiology of dyslipidemia and possible interaction with statin medications in our study. The keywords indicated above served as a valuable aid for choosing the appropriate publications, and the information gleaned from those articles helps to paint a complicated picture of the role that hypomagnesemia plays in the development of dyslipidemia and further in metabolic syndrome. As MetS is the most prevalent cardiovascular risk factor (CVRF), knowing the link between oxidative stress, diabetes mellitus, and cardiovascular disease (CVD), we expanded the investigation to additional pathological situations.

**Figure 1:**
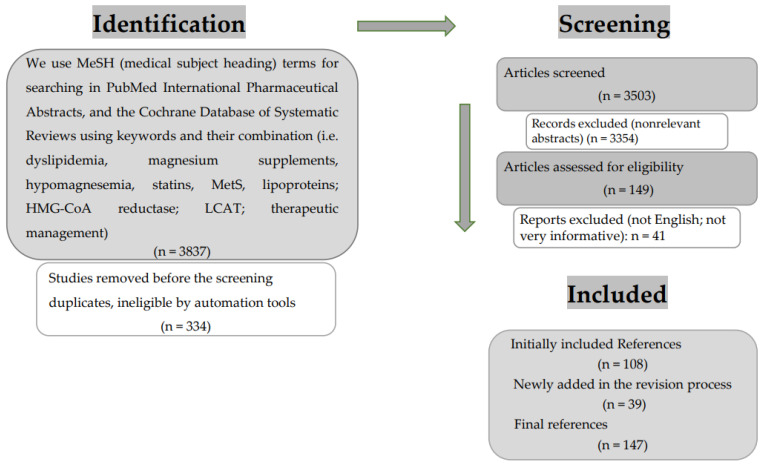
Studies identified via databases and registers.

Using keywords and their combination (i.e., dyslipidemia, magnesium supplements, hypomagnesemia, statins, MetS, lipoproteins; HMG-CoA reductase; LCAT; therapeutic management) were searched in PubMed, International Pharmaceutical Abstracts, and the Cochrane Database of Systematic Reviews. Meta-analyses, systematic reviews, and human clinical trials conducted in English were all included (Figure 1). The information that was not in English and the articles without pertinent findings were eliminated. A thorough investigation was conducted, new hypotheses were added, and a summary of the available data was produced.

Therefore, we conducted a meta-analysis of observational studies for the following purposes: (1) to evaluate the relationship between dietary magnesium consumption and dyslipidemia; (2) to assess the effect of dietary magnesium consumption on the risk of metabolic syndrome and its dose-response pattern; (3) to review the actual knowledge of statin medication, including positive effects and side effects; and (4) to examine the possible connection between Mg supplements and statin intake. 

## 3. General Considerations about the Metabolism of Plasmatic Lipoproteins

The blood transport of cholesterol esters and triglycerides is largely carried out with the help of lipoproteins, complex structures that contain hydrophobic components (triglycerides (TG) and esterified cholesterol (ES)), and, on the outside, have a hydrophilic coating made of phospholipids, free cholesterol, and proteins called apolipoproteins (Apo) (Table S1). There are five types of apoproteins (A, B, C, D, and E), some of which contain several subtypes [13]. Apoproteins contribute to the solubilization of LP particles in the aqueous plasma environment; they are activators of some enzymes involved in the lipoprotein (LP) metabolism (the lipoprotein lipase is activated by apo C-II and apo A-I activates LCAT) and are ligands for receptors involved in the cellular uptake of LP (apo B-100 is the ligand for the LDL receptor; apo E is the ligand for the uptake of remnant chylomicrons and IDL) [14,15]. In conclusion, apolipoproteins serve four main purposes: they are structural; they bind to lipoprotein receptors; they direct the synthesis of lipoproteins; and they either activate or inhibit the enzymes responsible for the metabolism of lipoproteins, as can be seen below in Table 1, a summary of the functions of the main apolipoproteins. Therefore, apolipoproteins are essential for the metabolism of lipoproteins.

Increased values of LDL cholesterol cause saturation of all the cells with cholesterol and a decreased number of receptors for LDL on the membrane (due to the phenomenon of downregulation). As a result, LDL particles persist longer in the plasma; some undergo oxidation by free radicals, resulting in oxidized LDL, which is no longer recognized by LDL receptors on the surface of hepatocytes and extrahepatic tissue cells [19]. Excess LDL and oxidized LDL will be captured by low-affinity scavenger receptors on the surface of monocytes/macrophages, receptors that are not regulated by the intracellular cholesterol content (they do not undergo the “downregulation” phenomenon) [17,18,19]. As a result, these cells are loaded with large amounts of cholesterol esters, turning into “foam cells” [22,23,24]. These cells accumulate in the arterial subendothelial space, where they initiate and maintain an inflammatory focus by attracting new monocytes and other inflammatory cells (lymphocytes) [24,25]. Migration into the subendothelial space and proliferation of smooth muscle cells in the medium wall of the arterial wall also occur. All these changes lead to the formation of the atheroma plaque, which has a lipid core and inflammatory cells inside, as well as areas of micro-thrombosis, and is covered with fibrous tissue on the outside [25,26,27]. Over time, this plaque causes the vascular wall to stiffen and the lumen to narrow, with decreased blood flow through the respective vessels (ischemia) and reduced oxygenation of the tissues irrigated by these vessels (tissue hypoxia) [20,23,25]. Under certain conditions, the fibrous hood of the plaque can break, and the contact of certain components inside the plaque with the plasma coagulation factors initiates the coagulation process, with the formation of a thrombus [25]. When the thrombus completely blocks the vascular lumen, the complete cessation of oxygen supply causes necrosis of the tissue irrigated by the respective vessel, a situation called infarction (depending on the location, it can be myocardial, cerebral, mesenteric infarction, etc.) [26,27,28].

**Figure 2:**
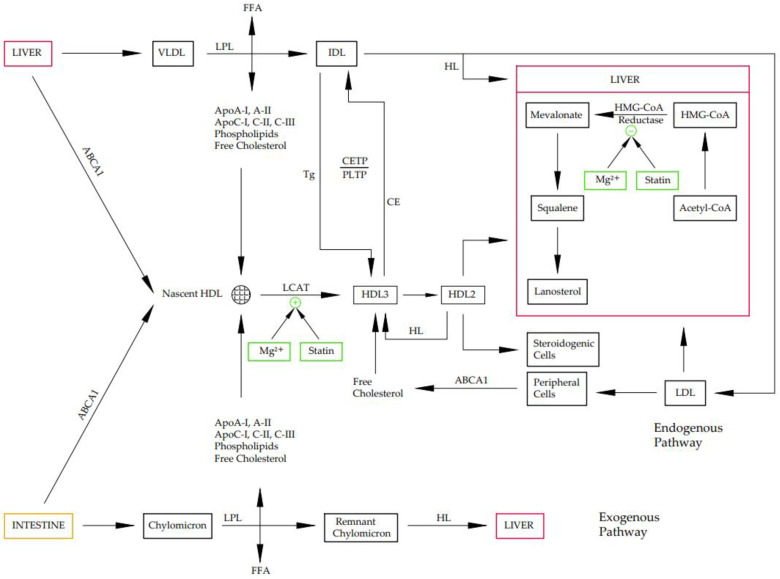
Cholesterol circuit: synthesis, its transport from the peripheral tissues to the liver, and its absorption at the level of intestinal epithelial cells. Abbreviation: ABCA1 = ATP-binding cassette transporter A1; Acetyl-CoA = Acetyl Coenzyme A; Apo = apolipoprotein; ApoA-I = Apolipoprotein A-I; ApoA-II = Apolipoprotein A-II; ApoC-I = Apolipoprotein C-I; ApoC-II = Apolipoprotein C-II; ApoC-III = Apolipoprotein C-III; CE = cholesteryl ester; CETP = cholesteryl ester transfer protein; FFA = free fatty acid; HL = hepatic lipase; HMG-CoA = 3-hydroxy 3-metylglutaryl coenzyme A; IDL = intermediate-density lipoprotein; LCAT = lecithin–cholesterol acyl transferase; LDL = low-density lipoprotein; LPL = lipoprotein lipase. Mg^2+^ = magnesium ions; PLTP = phospholipid transfer protein; TG = triglyceride; VLDL = very LDL.

Nascent HDLs (high-density lipoprotein cholesterol) have an enzyme produced by the liver called LCAT (lecithin–cholesterol acyl transferase) attached to their surface [28]. This enzyme, activated by its cofactor, apo A-I, catalyzes the esterification of cholesterol with a fatty acid transferred from the C-2 of lecithin [29,30,31] (Figure 2).

As can be seen in Figure 2, the resulting esterified cholesterol is entirely non-polar and migrates towards HDL in the center, forming a hydrophobic core → the HDL particle is changed and called HDL3 or mature HDL. As a result of the action of LCAT, the cholesterol content of the surface layer of HDL decreases [26,29,30,31]. As a result, HDL particles will be able to extract and remove excess cholesterol from extrahepatic tissues [29]. This transfer of cholesterol from tissues to HDL is mediated by a membrane protein called ABCA-1 (ATP-binding cassette transporter-1), which transports cholesterol using the energy resulting from ATP hydrolysis. Cholesterol extracted from tissues is esterified by LCAT and migrates to the core of the HDL particles, so they become larger and less dense and are called HDL2 [29,30,31,32,33,34]. HDL2 particles are transported to the liver, where they bind to receptors called SR-B1 (scavenger receptor class B1). The esterified cholesterol in the core of the particle is selectively taken up in the liver cell, but the HDL particle is not internalized but remains in the blood and becomes HDL3 again (following the decrease in cholesterol content), starting the cycle again. This transport of cholesterol from the extrahepatic tissues to the liver is known as the reverse transport of cholesterol (in the opposite direction to that carried out by LDL particles) [34]. It is particularly beneficial for vascular tissue: HDL reduces the risk of atherosclerotic plaque formation by reducing cholesterol levels in the arterial wall. In summary, the role of HDL particles is to take excess cholesterol from the extrahepatic tissues and transport it to the liver, where it will be eliminated from the body in the form of bile acids [29,34].

The VLDL metabolism is similar to that of chylomicrons. Nascent VLDL initially contains, in addition to lipids, apo B-100 [35,36]. After secretion by hepatocytes into the bloodstream, they receive apo C and apo E from HDL and become mature VLDL [36]. In the capillaries of some extrahepatic tissues, lipoprotein lipase (activated by apo C-II) degrades part of the TG from VLDL, with the resulting fatty acids being mostly captured in the respective tissues to be degraded as an energy source (e.g., in skeletal muscle, myocardium) or stored as TG (in adipose tissue). Subsequently, VLDL donates apo C back to the HDL particles [36,37]. The resulting particles are smaller and denser and are called remnant VLDL or IDL (intermediate-density lipoprotein). Compared to VLDL, IDL contain a smaller amount of TG and proportionally more cholesterol, and, among the apoproteins, they contain apo B-100 and apo E [34,35,36,37,38]. IDL particles have two possibilities of metabolism: on one hand, they are captured in the liver using receptors that recognize apo E on their surface (these are the same receptors that also capture residual chylomicrons (CM), the LDL receptor and LRP); the rest of the IDL particles will turn into LDL particles. In summary, the roles of VLDL particles are to provide fatty acids (synthesized by the liver) to the extrahepatic tissues and to be the precursors of LDL particles [33,39,40,41,42,43,44].

IDL particles that are not captured in the liver will undergo the following changes: (1)they will lose apo E to HDL particles (process mediated by cholesteryl ester transfer protein, CETP, and plasma phospholipid-transfer protein, PLTP); (2) this will be followed by increased PLTP activity (known to be a pro-atherogenic factor) and decreased CETP activity (recognized as a protective factor); (3) in the sinusoid capillaries of the liver, they undergo the action of hepatic lipase, which degrades most of the TG still contained in the IDL [45,46]. The resulting particles are LDL, which are rich in cholesterol (predominantly esterified), and contain very small amounts of TG, and the apoproteins contain only apo B-100. LDL will be taken up partly in the liver and partly in the extrahepatic tissues, via the LDL receptor, which recognizes apo B-100 as a ligand on the surface of LDL particles [37,38,39]. It should be noted that for VLDL to LDL conversions, a change in the spatial conformation of apo B-100 occurs, thus the recognition domain of the LDL receptor is exposed on the surface. As a result, the LDL receptor will be able to capture and internalize the LDL particles, a phenomenon called receptor-mediated endocytosis. (As previously mentioned, the hepatic LDL receptor also captures IDL particles, recognizing apo E on the IDL surface as its ligand; apo B-100 has, in these particles, a spatial conformation that does not allow recognition by the LDL receptor, the receptor binding not being exposed on the surface) [37]. Thus, the LDL receptor recognizes two ligands: apo E (present on the surface of remnant chylomicrons, CM, and intermediate-density lipoprotein, IDL), and apo B-100 on the surface of the LDL [37,38]. For this reason, it is also called the apo B-100/E receptor. A genetic deficiency of this receptor leads to the disease called familial hypercholesterolemia, transmitted autosomal dominantly and having an incidence of about 1:500 individuals in the general population [39,40,41,42]. It is manifested by a marked reduction in the capture of LDL particles, their excess accumulation in the plasma, and a significant increase in the risk of developing atherosclerosis and cardiovascular diseases [43,44].

LDL receptors in cell membranes bind LDL-forming complexes that pass through the cell by endocytosis to become endosomes. Later, the endosomes fuse with the lysosomes, forming endolysosomes, and the hydrolytic enzymes in the lysosomes degrade the components of the LDL particles: apoprotein B-100 is degraded into amino acids; cholesterol esterase hydrolyzes cholesterol esters to free cholesterol and fatty acids [37]. The LDL receptor is recycled to the plasma membrane to take up new LDL particles from the plasma [45]. The cholesterol reached intracellularly using LDL particles is used by cells to restore cellular and subcellular membranes; in the adrenal cortex and gonads, it is the precursor of steroid hormones, and, in the skin, it is the precursor of vitamin D3 [37,47]. The remaining cholesterol will regulate the intracellular cholesterol homeostasis in the following ways: (1) inhibition of the de novo synthesis of cholesterol in the cell, through the repression of HMG-CoA reductase (the regulatory enzyme of cholesterol synthesis) [34,37]; as a result, a cell receiving cholesterol from the plasma (via LDL) will reduce its local cholesterol synthesis [34,37,42,43,44,45]; (2) reducing the synthesis of new LDL receptors by decreasing the expression (repression) of the gene that codes for this receptor; as a result, the number of LDL receptors on the surface of the cells will decrease, preventing the capture of new LDL particles and thus preventing the overloading of the cell with cholesterol—a phenomenon known as “downregulation”; thus, the LDL receptor is a high-affinity but saturable receptor, the number of these molecules on the cell surface being dependent on the intracellular concentration of cholesterol [37,43,44,45]; (3) any cholesterol remaining that is more than current tissue needs is esterified under the action of an enzyme called ACAT (acyl-CoA cholesterol acyl transferase) and, temporarily, stored in the cytoplasm; ACAT is activated by cholesterol. In conclusion, the role of LDL particles is to transport cholesterol synthesized by the liver (and initially transported by VLDL) to the extrahepatic tissues, ensuring their cholesterol needs [29,37,47,48].

The progression to atherosclerosis, based on increased serum levels of LDL, begins with an altered balance between vasoconstriction and vasodilatation, damaged endothelium followed by increased endothelial permeability, and a cascade in the secretion of proinflammatory cytokines [49,50].

## 4. Role of Statins in Managing Dyslipidemia

Statins are HMG-CoA reductase inhibitors used in the management of hypercholesterolemia or familial dyslipidemias [51] and for primary or secondary prevention of atherosclerotic vascular disease (ASCVD) [52,53].

Hydroxymethylglutaryl-CoA (HMG-CoA) reductase inhibitors combined with diet and physical exercise are the common treatment for hypercholesterolemia, by reducing total cholesterol (TC), low-density lipoprotein cholesterol (LDL-C), and triglyceride (TG) levels. In the same time, they increase high-density lipoprotein cholesterol (HDL-C) concentrations [52].

### 4.1. Classification of Statins

Statins are classified based on their intensity or lipophilic or hydrophilic capacity.

The classification based on their intensity includes low-intensity statins (reduce LDL-C by less than 30%), moderate-intensity statins (reduce LDL-C by 30 to 50%), and high-intensity statins (reduce LDL-C by greater than 50%) [54]. The strongest statin is rosuvastatin, followed by pravastatin and atorvastatin in equivalent doses [55].

Statin classification includes lipophilic statins (simvastatin, atorvastatin, fluvastatin, lovastatin and pitavastatin) and hydrophilic statins (rosuvastatin and pravastatin) [56].

Lipophilic statins are able to pass the membranes and combine acyl chains. Lipophilic statins are distributed in different tissues due to their ability to circulate by passive diffusion. In the end, Cytochrome P450 (CYP) enzymes will metabolize statins. [57].

Hydrophilic agents (pravastatin and rosuvastatin) associated by polarity with the membrane use carriers to enter the cell and to inhibit the HMG-CoA reductase. That type of transportation is specific for the liver and is followed by a low potential of non-LDL effects at other sites. They are mostly eliminated without modification [58].

Statins lower plasma levels of total cholesterol, LDL-C, VLDL-C, triglycerides and increase plasmatic levels of HDL-C.

The biosynthesis of cholesterol has a very complex pathway, including the synthesis of mevalonate (MVA), the reduction of isopentenyl pyrophosphate (IPP) and dimethylallyl pyrophosphate (DMAPP), the synthesis of squalene, and the formation of cholesterol via lanosterol [59]. Statins intervene both during the synthesis of mevalonate by inhibiting (HMG-CoA) reductase and during the synthesis of squalene by inhibiting the formation of prenylated proteins (Figure 2).

### 4.2. Action of Statins on HMG-CoA

HMG-CoA reductase participates in the conversion of 3-hydroxy-3-methylglutaryl-CoA (HMG-CoA) to mevalonate in the hepatocytes. Statins bind to the active site of HMG-CoA reductase by competition and reduce its activity by inducing conformation changes. Statins have a more important binding affinity for HMG-CoA reductase than the substrate, so the synthesis of cholesterol in the hepatocytes will be reduced [52,58,60,61]. Second, the reduced synthesis of cholesterol will activate the sterol regulatory element-binding proteins (SREBP), followed by increased transcription of the LDL receptor in the hepatocytes. The plasmatic LDL and VLDL will be lowered after binding to the LDL receptors and will be processed by endocytosis in the hepatocytes [62,63].

Statins have also an important intrinsic cardiovascular protective effect, by inhibiting the synthesis of prenylated proteins (especially farnesyl and geranylgeranyl pyrophosphate) in the biosynthesis of cholesterol. By inhibiting the synthesis of isoprenoid intermediates, statins reduce the post-translational prenylation of small guanosine triphosphate-binding proteins. The following effectors (Rho kinase, nicotinamide adenine dinucleotide phosphate oxidases) will be also inhibited. The final result is a decreased proliferation of myocardial fibroblasts and inhibition of collagen proliferation [59,64,65].

The pleiotropic effect is due to four main mechanisms: plaque stabilization (by maintaining the fibrous structure of atherosclerotic plaque, lowering the macrophages, decreasing metalloproteinases of the matrix), reduced inflammation (by lowering the pro-inflammatory cytokines TNF-α, IL-6, IL-8), improved endothelial function (vasodilatation followed by better blood circulation due to increased endothelial nitric oxide synthase activity in the endothelial cells), and decreased thrombogenicity (by lowering the activity of platelets combined to reduced thromboxane A2 synthesis) [66,67,68,69].

### 4.3. Action of Statin over Lecithin–Cholesterol Acyltransferase (LCAT)

A glycoprotein called lectin–cholesterol acyltransferase (LCAT) functions as both a phospholipase A2 and an acyltransferase. It has a role in reverse cholesterol transport and is necessary for a balanced activity between all lipoprotein classes. It is produced by the liver and circulates reversibly bound to HDL and LDL in the plasma. On the surfaces of lipoproteins, lecithin–cholesterol acyltransferase catalyzes the esterification of free cholesterol. The most effective activator of LCAT, apo A-I, is found in HDL, which is the preferred substrate for it. The lipid content of HDL affects apo A’s capacity to activate LCAT; it is the most potent of the small HDL particles with a low phospholipid to apo A-I ratio [70,71,72].

The expansion of HDL particle cores is caused by the cholesteryl esters produced by LCAT. Although there is conflicting information regarding LCAT’s role in atherosclerosis development, it may represent a rate-limiting step in reverse cholesterol transport, making it a real point for therapy in atherosclerosis prevention. Injections using recombinant human LCAT (rhLCAT), cell and gene therapy, and the use of small chemical activators have all been tried with encouraging outcomes [73,74,75,76].

RhLCAT treatments have recently undergone promising clinical phase II trials. A new study demonstrated that overexpression of LCAT-improved LDL receptor-mediated RCT led to the regression of atherosclerosis and found MEDI6012 (one rhLCAT) as possible synergic with statins [77].

### 4.4. Administration of Statins

The recommendation for short-life statins (simvastatin, pravastatin, or fluvastatin) is to be taken in the evening, due to the fact that cholesterol synthesis happens at night during fasting [78]. Longer half-life statins (atorvastatin and rosuvastatin) can be administered in the morning or the evening daily at roughly the same time. As its absorption increases with food, lovastatin should be administered with meals [51,79].

Statins administration varies in specific patient population groups.

The statin metabolism is gender-dependent, so women have unique statin metabolic characteristics, due to lower glomerular filtration rates, higher body fat percentages, reduced muscle mass, overall faster statin metabolism and a higher risk of polypharmacy for older subjects or teratogenic effects for younger ones [80]. The effects and benefits of statin therapy are similar in men and women, but women receive its recommendation more rarely and in lower doses [81,82].

Subjects combining both statins and exercise have better results than those using only statin therapy regarding insulin sensitivity, exercise capacity, and lowering mortality [83].

In the elderly (>75 years of age) with clinically significant atherosclerosis and CVD, the advised therapy begins with moderate-intensity statins because of the increased side effects associated with high-intensity statins and a lower efficacy of metabolic mechanisms [84], although not all the studies show important rates of adverse age-related clinical events depending on the intensity of the statin [85].

Patients with chronic kidney disease should use atorvastatin, fluvastatin, pravastatin, or simvastatin because they do not undergo renal elimination, so they do not need dose adjustment [86]. Meanwhile, statins may improve renal function by lowering albuminuria [87,88,89].

Patients with compensated liver disease could use pravastatin and rosuvastatin, because compared to other statins, they are only partially metabolized by the liver. Statins are contraindicated in patients with acute liver failure or decompensated cirrhosis [87,90,91].

The most frequent side effect of statins is myalgia, affecting up to 10% of people taking them. Another, but less frequent, side effect of statin use is myositis. It presents an increase in creatine kinase (CK) [92]. The most dangerous adverse effect of statins, rhabdomyolysis is extremely rare (less than 1 per 100,000 patients treated annually), and it is linked to a substantial rise in CK (10 times higher than normal), abrupt renal failure related to myoglobinuria, electrolyte abnormalities and hemodynamic instability [92,93]. Because statins reduce the levels of coenzyme Q10 (ubiquinone) and other byproducts of the mevalonate pathway, necessary for skeletal muscle energy generation, they produce musculoskeletal toxicity [94]. Often, the symptoms appear a few weeks to months after therapy begins. After days to weeks of stopping the medicine, patients feel better, and their serum CK levels return to normal. The statins considered to have the least side effects on a muscular level are pravastatin and fluvastatin [92,95].

Statins can lead to higher levels of serum transaminases. If those values are three times higher than normal, the dose of statin must be reduced or the statin changed (preferably pravastatin) or switched to a different class of lipid-lowering drugs, although the liver enzymes levels usually improve regardless the interruption of medication [79,96].

Proteinuria, hematuria and rhabdomyolysis, as adverse effects following administration of high-intensity statins (such as rosuvastatin and simvastatin) may induce renal failure. In patients with renal alteration atorvastatin, fluvastatin, or pravastatin are recommended options [79].

The administration of high-intensity statins suppresses cholesterol synthesis and may trigger the onset of diabetes mellitus. Cholesterol is necessary for the formation of glucose transporter type 1, which facilitates the uptake of glucose into cells. The result of the process will be a higher plasma concentration of glucose [56]. The down-regulation of glucose transporter type 4 in adipocytes, reduced insulin signaling, and impaired Ca2+ signaling in pancreatic -cells are some of the mechanisms implicated in the development of type 2 diabetes mellitus. The impact of statins on epigenetics may also be a factor to induce diabetes mellitus through the differential expression of microRNAs [56]. Other data indicate that the diabetogenic effect of statins increases the risk of new-onset diabetes, especially in subjects with a “pre-diabetic” status [97].

Other side effects associated with statins have been reported in various case reports but they need more evidence data [84,85,86,87]. So far, lowering cholesterol drugs are not proven to increase malignancy, or neurological or respiratory symptoms [98].

## 5. Magnesium and Dyslipidemia

### 5.1. Magnesium Deficiency

Magnesium, the most common intracellular divalent cation, participates in around 300 enzymatic processes, including the metabolism of lipoprotein lipase (LPL), HMG-CoA reductase, and lecithin-cholesterol acyl transferase (LCAT), as well as the attenuation of Na-K ATPase and the breakdown of glycogen [99].

Just 1% of the body’s magnesium is found in circulation (0.3% in plasma/serum), while the majority of the body’s magnesium is found in the bony tissue (60%) and intracellular (40%) compartments [100]. Magnesium is distributed differently inside each individual compartment. For instance, bone has two deposits of magnesium: one in the bone network and the other present on the bone surface. Magnesium within cells is found in the cell membrane, intracellular components, and the nucleus in some amounts. The content of magnesium in red blood cells (RBC) is three times higher in blood than it is in plasma [100]. There are currently two recognized types of magnesium shortage: acute hypomagnesemia and chronic magnesium deficit. Extreme cramps, nystagmus, refractory hypokalaemia, refractory hypocalcemia, eclampsia during pregnancy and cardiac arrhythmias are only a few of the clinical signs and symptoms of extracellular acute hypomagnesemia [101]. In some instances, they respond quickly to intravenous magnesium. Although a chronic magnesium shortage is frequently coupled with normal magnesium levels in serum (0.75–0.95 mmol/L), which is mistakenly thought to eliminate the magnesium deficiency, it represents decreased levels of magnesium inside the cells and bone [100,101]. The overall amount of magnesium absorbed and eliminated through the kidneys, as well as factors such as food intake or supplementation and serum albumin level, can all affect the body’s magnesium levels [102]. Magnesium insufficiency is also made worse by other concomitant conditions such as inflammatory bowel disease, unmanaged diabetes, or renal disfunction [103,104]. The causes that produce magnesium deficiency are presented in Table 2**.**

**Figure 3:**
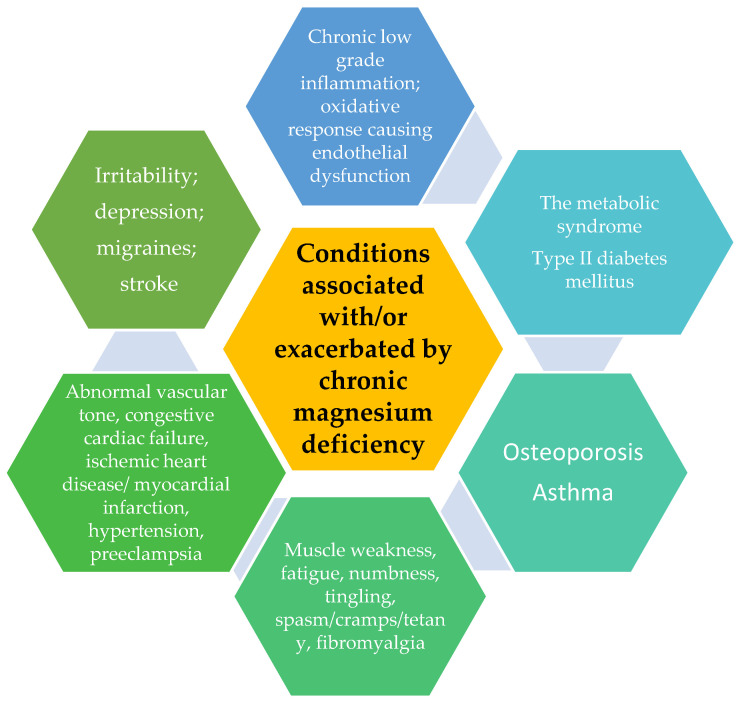
Pathologies connected to/or increasded by chronic magnesium deficiency.

However, numerous epidemiological, experimental, and clinical studies (not dependent of the type, design, measured indexes, size, and statistical approach of these studies) have demonstrated that chronic magnesium deficiency is connected with higher risk and prevalence of the pathologies listed in Figure 3.

Also, it was discovered that a magnesium deficit could predict negative outcomes, and when supplementation or treatment was started, there was a decreased chance of pathology [101].

### 5.2. Magnesium and MetS

According to the World Health Organization (WHO), coronary heart disease (CHD) accounts for more than 7 million deaths each year and is the leading cause of mortality in regardless the gender [133]. It is commonly recognized that type 2 diabetes (T2DM) is a significant risk factor for coronary heart disease (CHD). Patients with diabetes have twice the risk of developing coronary heart disease than people without the disease [104,134]. Several studies have demonstrated a link connecting CHD and metabolic syndrome (MetS), type 2 diabetes, and increased levels of oxidative and inflammatory stress biomarkers [135,136]. A complex biological process called oxidative stress is defined by an excessive creation of reactive oxygen species (ROS), which function as destabilizers of the body’s REDOX balance and cause oxidative disfunction. Oxidative stress impairs every process and even affects the equilibrium of nucleic acids. ROS interact with proteins and phospholipids, which causes structural alterations in tissues and organs. Those who are predisposed to heart problems typically develop a constellation of cardiovascular risk factors (CVRFs). Oxidative stress is frequently linked to cardiovascular diseases (CVDs) such as coronary artery disease (CAD), cardiomyopathy, or heart failure (HF), which may occur in people with hypertension (HTN), diabetes mellitus (DM), obesity, and other disorders [137]. According to some studies, patients with CHD and T2DM had significantly lower levels of magnesium [12,120,138,139,140]. There is also strong evidence linking magnesium deficiency to metabolic syndrome [107,135,141,142]. The elements of MetS, such as hyperglycemia, hypertension, hypertriglyceridemia, and insulin resistance, have been linked to hypomagnesemia [12]. The components of MetS, such as insulin sensitivity, fasting blood glucose (FBG), triglyceride (TG) levels, high-density lipoprotein cholesterol (HDL-C), and high blood pressure (BP), are said to respond favorably to magnesium supplementation [138]. The aforementioned metabolic illnesses all share the common pathophysiological feature of chronic low-grade inflammation, and magnesium deficiency can both directly and indirectly cause inflammation by altering the gut flora [108]. In addition, a lack of magnesium worsens immunological function by priming phagocytes, promoting granulocyte oxidative burst, activating endothelial cells, and raising cytokine production levels [12]. Magnesium has anti-inflammatory, glucose, and insulin metabolism-improving, endothelium-dependent vasodilation-improving, and lipid profile-normalizing properties [143]. C-reactive protein (CRP), a measure of systemic inflammation and a predictor of future cardiovascular events in individuals with MetS, and serum magnesium levels are inversely correlated [104,144]. At the same time, changes in the metabolism of several micronutrients were observed in obese patients, such as decreased magnesium concentrations in the serum, plasma, and erythrocytes [105,145,146]. Magnesium supplementation has been shown to have positive effects on patients with metabolic disorders in terms of their metabolic profile [109]. For example, Asemi et al. demonstrated that magnesium supplementation in pregnant women with gestational diabetes (GDM), in the form of magnesium oxide at a dose of 250 mg per day, significantly improved glycemic control, lipoproteins, and the biomarkers of oxidative stress and inflammation [109]. Higher magnesium intakes were associated with lower fasting insulin levels in healthy women without diabetes [147]. The relationship between total dietary magnesium intake and insulin responses to an oral glucose tolerance test was inverse [107,121].

Recent evidence from some meta-analysis studies demonstrated the efficacy of oral magnesium supplementation in significantly decreasing various inflammatory markers, especially CRP, and increasing nitric oxide (NO) [148]. The reduction of fasting blood glucose was also observed, as well as a 3–4 mm Hg reduction in systolic blood pressure (SBP) and 2–3 mmHg in diastolic blood pressure (DBP), with a beneficial impact on the lipid profile [99,106]. The experimental data show that magnesium deficiency worsens atherosclerosis while magnesium supplementation slows atherogenesis [11,149,150]. In addition, randomized controlled trials have shown that magnesium supplementation improves endothelial function while lowering blood pressure [110,111,118,124], arterial stiffness [151], fasting hyperglycemia [111,152], insulin resistance [153], and postoperative arrhythmias [150,154].

### 5.3. Magnesium–Statin-like Effect

Magnesium is crucial for regulating the activity of several key enzymes involved in lipid metabolism, including 3-hydroxy-3-methylglutaryl coenzyme A reductase (HMG-CoA reductase) [126,155], which controls cholesterol biosynthesis; lecithin–cholesterol acyltransferase (LCAT), which lowers plasma concentrations of LDL-c and VLDL-c [99,105]; desaturase (DS) [12,99]; and lipoprotein lipase (LPL) [127,156]. LPL, DS, and LCAT activity are suppressed by hypomagnesemia, and Mg supplementation can positively affect their expression [130,155]. On the other hand, Mg deficiency is associated with increased activity of HMG-CoA reductase [125]. The ratio of saturated to unsaturated fatty acids increases and the levels of TG, LDL, HDL, and VLDL decrease when the activity of this enzyme is impaired [157]. Modulation of the gene expression of LDLR (LDL receptor) and other transcription factors, such as sterol regulatory element-binding proteins SREBP-1a and SREBP-2, may also contribute to the hypercholesterolemic effect of an inadequate intake of Mg, although the increase in LDL concentration is mediated by increased LDLR and SREBP expression [158]. According to a study in type 1 diabetes mellitus (T1DM) patients, the Mg levels and blood levels of oxidized low-density lipoprotein (ox-LDL) are linked [12]. Magnesium also seems to affect the expression of genes that regulate a number of processes, including adipogenesis, lipolysis, and inflammation, such as peroxisome proliferator-activated receptor gamma (PPARγ) [105,159]. Also, research has shown that a dietary magnesium deficiency causes an higher activity of the enzyme’s serine palmitoyl CoA transferase 1 and 2, which, in turn, triggers the processes of atherogenesis, angiogenesis, and immunoreaction [12,105,160].

A 12-week randomized, double-blind, placebo-controlled clinical trial comparing the effects of magnesium oxide (250 mg/day) versus placebo on anthropometric indices, blood pressure, blood glucose, insulin, C-reactive protein, uric acid, and a lipid profile was performed on individuals with prediabetes (n = 86). Compared to the placebo group, subjects who took magnesium supplements had significantly increased levels of HDL cholesterol at the end of the study. However, magnesium supplementation at the dose and time mentioned above did not alter other cardiometabolic parameters [161].

Participants with moderate coronary artery disease (CAD) lowered their serum levels of LDL-C, SGOT, SGPT, and ox-LDL by taking 300 mg/day of MgSO4 for six months in a double-blind, randomized clinical investigation [146].

In addition, a recent meta-analysis of studies that included people with hypertriglyceridemia revealed an important decrease in triglyceride levels after magnesium supplementation, indicating a potential beneficial effect of magnesium supplementation on dyslipidemic diseases [99].

Clinical research using oral Mg supplementation provides the majority of the evidence for relationships between Mg and the blood lipid profile [112,113,126,156,157,162,163,164,165,166]. The authors of various meta-analyses describing this link stressed the inconsistency of the findings of this research [167,168]. The significant variety of groups examined, the numerous approaches to measuring blood serum Mg concentration, and the absence of stratification of the subgroups of patients compared have all contributed to the ambiguity of the results produced in this sector [12].

## 6. Statin vs. Magnesium Comparison for Cholesterol Control

A comparison of Mg effects on lipoproteins with those of statin medication is necessary because Mg^2+^-ATP is the regulating factor for the rate-limiting enzyme in the cholesterol production sequence which the statin pharmaceuticals target. A sequence of enzymatic processes that change HMG-CoA into cholesterol results in the synthesis of cholesterol in the circulation, as well as the cholesterol needed for hormone production and membrane repair. The enzymatic conversion of HMGCoA to mevalonate via HMGCoA reductase is the pathway rate-limiting step. Mg and statins both block the enzyme. Major trials have repeatedly demonstrated that statins lower cholesterol blood levels by 35 to 65% when taken by patients with high LDL-cholesterol (LDL-C) values [122,169,170,171,172,173]. Moreover, they lower the frequency of heart attacks, angina, other nonfatal cardiac events, and cardiac, stroke, and overall mortality. These statin effects come more from lowering mevalonate formation than from lowering LDL-C. Mg has effects that are similar to statin effects, such as improving endothelial function, inhibiting vascular smooth muscle cell and macrophage proliferation and migration, promoting plaque stabilization and regression, and lowering inflammation. For instance, Mg is required for the enzyme that inactivates HMG-CoA reductase, transforming it in a modulater rather than an inhibitor of Reductase. Lecithin–cholesterol acyl transferase (LCAT), which lowers triglyceride and LDL-C levels and boosts HDL-C levels, also needs magnesium to function. Statins have no direct effect on desaturase, another Mg-dependent enzyme involved in lipid metabolism.

A 4-week treatment with oral Mg supplementation (Mg (OH)2: 411–548 mg Mg/day) or a placebo was given to 33 participants in double-blind, placebo-controlled research published in 1997 in the British Journal of Nutrition [122]. The urine excretion of Mg considerably grew in the first two weeks and the next few weeks of Mg supplementation, and the urinary excretion of Na also significantly increased throughout the experimental period. Apolipoprotein AI, HDL cholesterol, and lecithin–cholesterol acyltransferase (LCAT) all showed statistically significant increases after the Mg supplementation. During the experimental period, a positively significant connection between the levels of LCAT and urine Mg excretion was shown (displayed as a percentage of the run-in value). When compared to the first two weeks and the run-in periods, the total cholesterol:HDL cholesterol ratio significantly decreased over the final two weeks of the magnesium supplementation, but this did not happen in the placebo group. These findings imply that Mg supplementation may improve serum lipids in humans by activating LCAT while reducing blood pressure by suppressing adrenergic activity and possibly natriuresis [122,169,170,171,172]. Statins have different effects from those produced by naturally occurring magnesium levels or by taking magnesium supplements, as shown by A. Rosanoff and her colleagues in a study published in Nutrients, in 2021 [172].

A study that was published in 2014 examined how patients with hyperlipidemia responded to using magnesium supplementation in addition to atorvastatin. This study’s major goal was to clarify if taking oral magnesium supplements could prevent or delay the myalgia that statins cause. According to the findings, patients who received atorvastatin along with a magnesium treatment had significantly increased levels of serum magnesium, plasma L-CAT, and HDL cholesterol, as well as significantly decresed levels of total cholesterol, LDL cholesterol, and triglycerides as well as non-significantly decreased levels of CK compared to patients who received atorvastatin alone [173]. Finally, magnesium supplements work better than atorvastatin alone at improving all-lipid profiles and controlling dyslipidemia. The Mg supplement to atorvastatin may delay and offer some protection against statin-induced myopathy, which, in turn, may boost patient compliance, but it does not prevent the rise of CK [173].

Another study published in 2021 examined the potential for treating HFD-induced bone loss in mice with a combination of Mg^2+^ and simvastatin (SIM) and the underlying mechanisms. Simvastatin and magnesium work together synergistically to reduce bone loss caused by a high-fat diet. Their formulation might serve as a more affordable alternative therapy for obesity-related bone loss [128]. Lecithin–cholesterol acyl transferase (LCAT), that decreases LDL and triglyceride levels and increases HDL-cholesterol levels, is activated by magnesium, which also targets the enzyme HMG CoA reductase [72,73,74,75,105]. Additionally, magnesium activates desaturase and other crucial enzymes involved in lipid metabolism, which are not directly affected by statins. Omega-3 linoleic acid and omega-6 linolenic acid, which are necessary fatty acids, are first converted into prostaglandins by the enzyme desaturase [105,118]. Prostaglandins, like prenylated proteins, have a domino effect of physiological actions that are essential for cardiovascular and general health.

Although there are several enzymes involved in the metabolism of lipids and cholesterol, HMG CoA reductase is a key one. In a dosage–response relationship, statins work by temporarily inhibiting the enzyme, whereas the magnesium ion (Mg^2^) is a crucial component of the intricate control and regulation of this crucial process [105,118,122].

While some statins can increase HDL levels and reduce triglycerides, Mg supplements reliably lower LDL cholesterol [128,169,170,171,172,173,174].

While Mg supplements tend to protect against myopathy and have only brief diarrhea or moderate gastrointestinal distress as a side effect, statins elevate hepatic enzymes, may lead to myopathy, and have many additional side effects [116,119,123,131,175,176,177,178] (Table S1). Statins and normal Mg levels work both to prevent blood coagulation, reduce inflammation, and prevent atherogenesis formation [114,115,117,118,129,132,179].

## 7. Conclusions

Magnesium supplements have complementary and parallel effects to those of statin medication. Both inhibit the HMG-CoA reductase enzyme, which is responsible for converting HMG-CoA to mevalonate, the first step in the production of cholesterol.

Mg triggers a desaturase, which turns 3- and 6-omega fatty acids into prostaglandins, as well as LCAT (lecithin–cholesterol-acyl-transferase), an enzyme that lowers TG and LDL-C levels and boosts HDL-C levels.

Similar to statins, Mg has functions that are crucial to cardiovascular and general health. The association between Mg supplementation and atorvastatin is able to postpone and offer some protection for myopathy as a side effect induced by statins, which, in turn, may boost patient compliance, but it does not prevent the rise of CK.

Most clinical trials have supported the positive impact of magnesium supplementation and diet in the context of metabolic diseases. Targeting Mg homeostasis may constitute a new strategy to prevent and treat metabolic diseases and their repercussions, even though the precise mechanisms of Mg action are still unknown. Further randomized controlled trials examining Mg supplementation methods are nevertheless required.

## Figures and Tables

**Table 1:** Enzymes involved in plasma lipoprotein metabolism.

Apolipoprotein	Lipoprotein Association	Function	References
ApoA-I	HDL	Triggers LCAT; controls ABC transporter	[13,14,15,16,17]
ApoA-II	HDL	Structural protein for HDL, Activates hepatic lipase	[14,18,19]
ApoA-IV	Chylomicrons, HDL	Unknown	[14,19,20,21,22]
ApoB-48	Chylomicrons	Structural protein for chylomicrons	[13,14,15,16,17]
ApoB-100	VLDL, LDL	Binds to LDL receptor	[17,18]
ApoC-I	VLDL, LDL	Activates LCAT	[14]
ApoC-II	Chylomicrons, VLDL, LDL	Activates lipoprotein lipase	[13,14,15]
Apo C-III	Chylomicrons, VLDL, LDL	Inhibits lipoprotein lipase	[13,15,18]
ApoD	HDL	Unknown (has been proposed to play a role in stabilizing LCAT and increasing cholesterol esterification)	[14]
ApoE	Chylomicrons, VLDL, LDL	Triggers clearance of VLDL and chylomicron remnants	[13,14,15,22]

**Table 2:** Causes of magnesium deficiency.

Causes of Magnesium Deficiency	
**Insufficient intake** A diet low in Magnesium [102,105] Slimming cures [102,106]	**Intensification of losses** At the gastrointestinal level: vomiting; purgatives/laxatives; trailing diarrhea [103,107,108] By the renal route: nephropathies; diuretics; chronic alcoholism; diabetes [104,109,110,111,112,113,114,115]
**Decreased intestinal absorption** Conditions after intestinal resection [103,116] Diarrhea [103,107,108] Malabsorption [103,107,108,117] Chron’s disease [103,108,117] Ulcerative colitis [103,117] Coeliac disease [103,107]	**Stressing Factors** Pregnancy [32,34,102,118,119] Lactation [32,34,102,118,119] The growing period [102,120] Sport performance [102,121,122,123] Old age [103,107,109,119,124,125] Convalescence [86,87,102]
**Endocrine disorders** Hyperthyroidism [104,126,127] Aldosteronism [104,126,127] Hyperparathyroidism [104,126,127,128] Poorly controlled diabetes [104,109,129]	**Interference of absorption** Increased calcium intake [102,117,128] Hyper protein diet [102,108] Lipid foods [102,128,130,131] Excessive alcohol consumption [102,119,132]

## Data Availability

Not applicable.

## Supplementary Materials

The following supporting information can be downloaded at: https://www.mdpi.com/article/10.3390/cimb45040205/s1, Table S1: Research Investigating the Interaction Between Serum Lipids and Magnesium
