# Supplementary material for: The Link between Magnesium Supplements and Statin Medication in Dyslipidemic Patients
Source: Curr Issues Mol Biol. 2023 Apr 5;45(4):3146–67. doi: 10.3390/cimb45040205 (PMC10136538; doi:10.3390/cimb45040205)
Supplement: Supplementary file 1 [file cimb-45-00205-s001.zip › cimb-2280636-supplementary.pdf]

Table S1. Research Investigated the Interaction Between Serum Lipids and Magnesium.

No. Crt.	Study	Type	Population	Conclusion
163	El-Haggar Sahar, M.; Mostafa Tarek, M. Role of Magnesium Supplement on Hyperlipidemia and L-CAT Level in Patient on Atorvastatin Therapy. British Journal of Pharmaceutical Research. 2014 June; 4(12): 15211534.	prospective cohort study	40	Mg++ supplement to atorvastatin improve all lipid profile and provide better control on dyslipidemia than atorvastatin alone
168	Jiao, Y.; Li, W.; Wang, L.; Jiang, H.; Wang, S.; Jia, X.; Wang, Z.; Wang, H.; Zhang, B.; Ding, G. Relationship between Dietary Magnesium Intake and Metabolic Syndrome. Nutrients. **2022** May 11;14(10):2013. doi: 10.3390/nu14102013.	longitudinal follow-up study initiated in 1989 that had completed 11 waves of followup	6104 subjects adults aged 18 years and above who participated in at least two follow-up surveys in 2009, 2015 and 2018	overall and non-linear associations between dietary magnesium and MetS and its components were statistically significant, the risk of them decreased significantly when magnesium intake was lower than 280 mg/day, and then the curve leveled off or slightly increased.
169	Jin, S.; Liu, J; Jia, Y.; Han, T.; Zhao, X.; Sun, C.; Na, L. The association of dietary flavonoids, magnesium and their interactions with the metabolic syndrome in Chinese adults: a prospective cohort study. Br J Nutr. **2021** Sep 28;126(6):892902. doi: 10.1017/S0007114520004754.	prospective cohort study	6417 participants aged 20 to 74 years from the Harbin Cohort Study on Diet, Nutrition and Chronic Noncom-municable Diseases	higher intake of flavonoids was associated with a decreased risk of the MetS, which was more significant in the presence of higher Mg intake. In addition, higher intake of flavonoids was also associated with a de-creased risk of central obesity.
170	Yang, N.; He, L.; Li, Y.; Xu, L.; Ping, F.; Li, W.; Zhang, H. Reduced Insulin Resistance Partly Mediated the Association of High Dietary Magnesium Intake with Less Metabolic Syndrome in a Large Chinese Population. Diabetes Metab. Syndr. Obes. **2020**; 13:2541–2550. doi: 10.2147/DMSO.S257884.	cross-sectional study	8120 participants	Significant and independent negative relation-ship among weight-adjusted dietary magnesium intake, HOMA-IR, and MetS in a large Chinese population. IR partly mediated the relationship between dietary magnesium intake and MetS.
13	Castellanos-Gutiérrez, A.; Sánchez-Pimienta, T.G.; Carriquiry, A.; da Costa, T.; Ariza ,A.C. Higher dietary magnesium in-take is associated with lower body mass index, waist circumference and serum glucose in Mexican adults. Nutr. J. **2018**; 17:114. doi: 10.1186/s12937-0180422-2. [	a population-based multistage study	1739 participants	The results of this study suggest that in-creased dietary magnesium intake is associ-ated with lower BMI and WC. In women without impaired glucose me-tabolism, it is also associated with lower serum glucose concentrations. These results highlight the importance of an adequate dietary magnesium intake

171	Jin, H.; Nicodemus-Johnson, J. Gender and Age Stratified Analyses of Nutrient and Dietary Pattern Associations with Circulating Lipid Levels Identify Novel Gender and Age-Specific Correlations. Nutrients. **2018** Nov 14;10(11):1760. doi: 10.3390/nu10111760.	Retrospective National Health and Nutrition Examination Study (NHANES) 2001–2013	Adult individuals n-12,284	Dietary Mg was positively correlated with HDL concentration but negatively with the TC/HDL-C ratio in females
172	Bain, L.K.; Myint, P.K.; Jennings, A.; Lentjes, M.A.; Luben, R.N.; Khaw, K.T.; Wareham, N.J.; Welch, A.A. The relationship between dietary magnesium intake, stroke and its major risk factors, blood pressure and cholesterol, in the EPIC-Norfolk cohort. Int J Cardiol. **2015** Oct 1; 196:108-14. doi: 10.1016/j.ijcard.2015.05.166.	cross-sectional study (European Prospective Investigation into Cancer (EPIC)-Norfolk)	Adult individuals (40–75 years) n-4443	Inverse relationship between high dietary Mg intake (mean 456 mg/d) and serum TC (p-trend = 0.02 men and 0.04 women)
173	Yamori, Y.; Sagara, M.; Mizushima, S. et al. An inverse association between magnesium in 24-h urine and cardiovascular risk factors in middle-aged subjects in 50 CARDIAC Study populations. Hypertension Research: Official Journal of the Japanese Society of Hypertension. **2015** Mar;38(3):219-225. DOI: 10.1038/hr.2014.158.	Cross-sectional analysis (World Health Organizationcoordinated Cardiovascular Diseases and Alimentary Comparison (CARDIAC) Study (1985–1994)	Adult participants (48–56 years) n-4211	Mg/creatinine (Cre) ratio was inversely associated with BMI, SBP, DBP, and TC (p for linear trend < 0.001 for each)
139	Rotter, I.; Kosik-Bogacka, D.; Dołęgowska, B.; Safranow, K.; Karakiewicz, B.; Laszczyńska, M. Relationship between Serum Magnesium Concentration and Metabolic and Hormonal Disorders in Middle-Aged and Older Men. Magnes. Res. **2015**; 28:99–107. doi: 10.1684/mrh.2015.0391.	prospective cohort study	313 men aged 5075 years	(+) correlations: Mg-TC (r = 0.25, p < 0.0001), Mg-LDL-C (r = 0.26, p < 0.0001)
174	Yuan, Z.; Liu, C.; Tian, Y.; Zhang, X.; Ye, H.; Jin, L.; Ruan, L.; Sun, Z..; Zhu, Y. Higher Levels of Magnesium and Lower Levels of Calcium in Whole Blood Are Positively Correlated with the Metabolic Syndrome in a Chinese Population: A Case-Control Study. Ann. Nutr. Metab. **2016**; 69:125–134.	Case-control study	408 patients	(+) correlations: Mg-BMI (r = 0.128, p < 0.05), Mg-TC (r = 0.254, p < 0.05), Mg-LDL-C (r = 0.280, p < 0.05)

175	Dibaba, D.T.; Chen, C.; Lu, L.; Bidulescu, A.; Fly, A.D.; Xun, P.; Judd, S.E.; Cushman, M.; Kahe, K. Magnesium intake is inversely associated with the risk of metabolic syndrome in the Reasons for geographic and racial differences in stroke (REGARDS) cohort study. Clin Nutr. **2021** Apr;40(4):2337-2342. doi: 10.1016/j.clnu.2020.10.024.	Population-based cohort study	n=30,239 aged ≥45 years at baseline, and self-identified as black or white	A total of 1,470 participants developed MetS during an average follow-up of 10 years. Comparing the highest quintile of total Mg intake (>437.9 mg/day) to the lowest group (<223.5 mg/day), total Mg intake had a significant inverse association with the risk of MetS [relative risk (RR) = 0.79 (0.63, 0.98), P trend = 0.043]. Dietary Mg intake was inversely associated with MetS [RR = 0.72 (0.56, 0.91), P trend = 0.006]. Adjusting for baseline components of MetS attenuated the associations, but the linear trends remained.
176	Dominguez, L.J.; Gea, A.; RuizEstigarribia, L.; Sayón-Orea, C.; Fresán, U.; Barbagallo, M.,;RuizCanela, M.; Martínez-González, M.A. Low Dietary Magnesium and Overweight/Obesity in a Mediterranean Population: A Detrimental Synergy for the Development of Hypertension. The SUN Project. Nutrients. **2020** Dec 31;13(1):125. doi: 10.3390/nu13010125.	Prospective cohort study	14,057 participants of the SUN (Seguimiento Universidad de Navarra)	Dietary magnesium intake < 200 mg/d was independently associated with a higher risk of developing hypertension in a Mediterranean cohort, stronger for overweight/obese participants.
177	Toprak, O.; Sarı, Y.; Koç, A.; Sarı, E.; Kırık, A. The Impact of Hypomagnesemia on Erectile Dysfunction in Elderly, Non-Diabetic, Stage 3 and 4 Chronic Kidney Disease Patients: A Prospective Cross-Sectional Study. Clin. Interv. Aging. **2017**;12:437–444.	Prospective Cross-Sectional Study.	372 patients	hypoMg: ↑ obesity ($p = 0.003$), ↑ MetS ($p = 0.026$), ↓ HDL-C ($p = 0.009$)
178	Dey, R.; Rajappa, M.; Parameswaran, S.; Revathy, G. Hypomagnesemia and Atherogenic Dyslipidemia in Chronic Kidney Disease: Surrogate Markers for Increased Cardiovascular Risk. Clin. Exp. Nephrol**. 2015**; 19:1054–1061. doi:	Prospective cohort study	180 patients	↓ serum and urinary Mg<br>↑ TC, LDL-C, non-HDL-C ($p < 0.001$ for all)<br>(+) correlation of serum Mg and HDL-C ($r = 0.326$, $p = 0.002$)<br>(−) correlations of serum Mg and TC ($r = −0.247$, $p = 0.019$), LDL-C ($r = −0.303$, $p = 0.004$), non-HDL-C ($r = −0.289$, $p = 0.006$), Framingham risk score ($r = −0.939$, $p < 0.001$), the presence of MetS ($r = −0.830$, p <

	10.1007/s10157-015-1097-z.			0.001), CKD severity (r = −0.245, p = 0.02)
179	Cambray, S.; Ibarz, M.; Bermudez-Lopez, M.; Marti-Antonio, M.; Bozic, M.; Fernandez, E.; Valdivielso, J.M. Magnesium Levels Modify the Effect of Lipid Parameters on Carotid Intima Media Thickness. Nutrients. **2020**; 12:2631. doi: 10.3390/nu12092631.	Prospective cohort study	1754 patients	↑ CKD severity = ↑ Mg and ↑ TG (p < 0.001 for trend)<br>↑ CKD severity = ↓ TC, LDL-C, HDL-C (p < 0.001 for)<br>associations of Mg with T2DM (r = −0.070, p = 0.003), HTN (r = 0.053, p = 0.028), BMI (r = −0.053, p = 0.027)<br>association of CIMT and Mg-TC (β = 0.008, SE = 0.003, p = 0.011), Mg-HDL-C (β = −0.007, SE = 0.003, p = 0.016), Mg-LDL-C (β =
				−0.007, SE = 0.003, p = 0.03), Mg-TG (β = −0.0014, SE = 0.0005, p = 0.01) interactions
123	Hamedifard, Z.; Farrokhian, A.; Reiner, Ž.; Bahmani, F.; Asemi, Z.; Ghotbi, M.; Taghizadeh, M. The Effects of Combined Magnesium and Zinc Supplementation on Metabolic Status in Patients with Type 2 Diabetes Mellitus and Coronary Heart Disease. Lipids Health Dis. **2020**; 19:112. doi: 10.1186/s12944-020-01298-4.	randomized, double-blind, placebo-controlled trial	60 patients	HDL-C: ↑<br>TC, TC/HDL-C, LDL-C, VLDL, TG: no effect

143	Rashvand, S.; Mobasseri, M.; Tarighat-Esfanjani, A. Effects of Choline and Magnesium Concurrent Supplementation on Coagulation and Lipid Profile in Patients with Type 2 Diabetes Mellitus: A Pilot Clinical Trial. Biol. Trace Elem. Res. **2020**; 194:328–335. doi: 10.1007/s12011019-01802-7.	randomized, double-blind, placebo-controlled trial	96 patients	Compared with baseline values, there were significant differences in serum magnesium, HDL, and triglycerides (TG) following cholinemagnesium co-supplementation ($p < 0.05$); however, there were no significant differences in serum magnesium, HDL, and TG among the groups ($p > 0.05$). Overall, concurrent supplementation of magnesium and choline is more effective than either magnesium or choline alone to improve coagulation in subjects with T2DM.

Abbreviation: Mg, magnesium. MetS, metabolic syndrome. T2DM, type 2 diabetes mellitus. HTN, hypertension. hypoMg, hypomagnesemia. OR, odds ratio. CI, confidence interval. ↑, increased. ↓, decreased. (+), positive. (−), negative. HDL-C, high-density lipoprotein cholesterol. LDL-C, low-density lipoprotein cholesterol. TC, total cholesterol. TG, triglycerides. VLDL, very low-density lipoprotein cholesterol. apoA1, apolipoprotein A1. RTC, randomized controlled trials.
